# Long-term efficacy of glycopyrrolate on sialorrhea in Goldenhar syndrome: a case report

**DOI:** 10.1186/s13052-022-01320-8

**Published:** 2022-09-06

**Authors:** Gessica Della Bella, Enrico Castelli, Federico Vigevano

**Affiliations:** 1 Intensive Neurorehabilitation and Robotics Department – Bambino Gesù Paediatric Hospital, Via Torre di Palidoro, 00054 Passoscuro-, Fiumicino, Italy; 2 Neurosciences Department, Bambino Gesù Paediatric Hospital, Piazza di Sant’Onofrio 4, 00165 Rome, Italy

**Keywords:** Sialorrhea, Glycopyrronium oral solution, Goldenhar Syndrome, Drooling Impact Scale, Modified Teachers’ Drooling Scale

## Abstract

**Background:**

Goldenhar syndrome (GS) is a rare congenital disease characterized by impaired development of different facial structures and deformations of the teeth structures. Sialorrhea, which can cause difficulties in breathing and language impairment, is very common in GS and often difficult to treat. This case report highlights the short- and long-term importance of the therapeutic choice – glycopyrronium in oral solution - for the treatment of sialorrhea in children with poly-malformative syndrome, complicated by outcomes of post-hemorrhagic hydrocephalus.

**Case presentation:**

We report the case of a 6-year-old child with GS, carrying a percutaneous endoscopic gastrostomy after tracheostomy. The child also presented developmental dysfunction of oral motor skills of feeding, complicated by severe sialorrhea, related to the maxillo-facial dysmorphism. Sialorrhea caused several respiratory tract infections and led to an increase in the care burden. Both the inoculations of botulinum toxin and the treatment with scopolamine transdermal patch have shown mild and transient efficacy. The therapeutic choice of glycopyrronium in oral solution was the most suitable for this patient, leading to long-term sialorrhea control.

**Conclusions:**

This clinical experience represents the first long-term efficacy and tolerability evaluation in using glycopyrrolate oral solution in treating drooling in children with GS. The reduction of drooling over time and the lack of clinically relevant adverse events have contributed to the decrease of respiratory tract infections, the development of oral motor skills, and determining a positive psycho-social impact on the patient's quality of life and her family.

## Background

Goldenhar syndrome (GS) is a rare congenital disease characterized by impaired development of different facial structures and deformations of the teeth structures, arising from defects in the first and second brachial arches [1].

Due to craniofacial abnormalities, and mainly to unilateral facial microsomia, patients with GS may present mechanical obstruction of the upper airway. Use of nasopharyngeal cannulas and, in severe cases, tracheostomy at birth is required as a standard procedure for airway control [1–3]. Sialorrhea, which can cause difficulties in breathing and language impairment, is very common and often difficult to treat.

This case report highlights the short- and long-term importance of the therapeutic choice – glycopyrronium in oral solution [4] for the treatment of sialorrhea in children with poly-malformative syndrome, complicated by outcomes of post-hemorrhagic hydrocephalus.

## Case presentation

We report the case of a 6-year-old child with GS, carrying a percutaneous endoscopic gastrostomy after tracheostomy. Due to her maxillo-facial dysmorphism (right massive facial hypoplasia) and respiratory issues (first-degree supra-cannular tracheal stenosis), she was followed from birth by another specialized hospital. The child also presented developmental dysfunction of oral motor skills of feeding, complicated by severe sialorrhea, related to the maxillo-facial dysmorphism. From the age of 3 years, the patient was followed by the Dysphagia Service of our hospital. She carried out intensive training cycles of speech therapy. The severity of sialorrhea caused several respiratory tract infections and led to an increase in the care burden, with a high number of aspirations per day and frequent bib changes. For these reasons, from the age of 3 years, the patient was treated with botulinum toxin type A inoculations in the salivary glands with poor outcomes. In April 2018 (after 1 year), the patient was treated with a scopolamine transdermal patch, applied to the latero-cervical/auricular area. The initial dose, half patch (0.75 mg) every 72 hours, resulted in only a partial reduction of saliva production. Three months later, in July 2018, the dose was increased to 1 whole patch (1.5 mg) every 72 hours, but no improvement of drooling was reported. Therefore, in November 2018, the patient was treated with glycopyrronium oral solution 0.32 mg/ml (equivalent to 0.4 mg/ml glycopyrronium bromide), with the following regimen: 0.480 μg glycopyrronium (1.5 ml) twice a day (0.960 μg /day; dosage level 2 out of 5, according to the SmPC) [4].

After 7 months, at the first follow-up visit (June 2019), the third dose of 0.480 μg/day was added to the therapeutic scheme. In January 2020, because of weight gain and a slight increase in drooling, the dosage was adjusted to 0.960 μg × 3/day (dosage level 3 out of 5, according to the SmPC) [4]. The severity and frequency of drooling were assessed with the Drooling Impact Scale (DIS) [5] and the modified Teachers' Drooling Scale (mTDS) [6] as outcome measures submitted to the parents.

The improvement obtained was significant and confirmed by the decrease in the DIS and mTDS scales scores. At the last visit, in January 2021, 2 years and 2 months after the onset of treatment, the good efficacy was confirmed (Table 1). The patient did not present the side effects of anticholinergic drugs, whose most common side effects include constipation, flushing pupillary dilation/visual disturbance, and urinary retention.

**Table 1:** Drooling assessment with DIS score and mTDS score during the different time point of the therapy

Evaluation periods for sialorrhea before and after treatment with oral glycopyrronium solution	DIS score	mTDS score
November 2018, initial evaluation before therapy	79	8
January 2020, evaluation after more than 12 months from the beginning of the therapy	29	2
January 2021, evaluation after 26 months from the beginning of the therapy	27	2

## Discussion and conclusions

This case report highlights the difficulty of managing sialorrhea in children with challenging clinical rehabilitation, and specifically with feeding dysfunctions. In our patient, both the inoculations of botulinum toxin and the treatment with scopolamine transdermal patch have shown mild and transient efficacy. The therapeutic choice of glycopyrronium in oral solution was the most suitable for this patient, leading to long-term sialorrhea control.

This clinical experience currently represents the first long-term efficacy and tolerability evaluation in using glycopyrrolate oral solution in treating drooling in children with GS. Even if a greater sample should be considered to draw more solid conclusions, in this case the reduction of drooling over time and the lack of clinically relevant adverse events have contributed to the decrease of respiratory tract infections, the development of oral motor skills, and determining a positive psycho-social impact on the patient's quality of life and her family.

## Data Availability

Not applicable

## Abbreviations

GS: Goldenhar syndrome
DIS: Drooling Impact Scale
mTDS: Modified Teachers’ Drooling Scale
